# Efficacy of sorafenib on metastatic renal cell carcinoma in Asian patients: Results from a multicenter study

**DOI:** 10.1186/1471-2407-9-249

**Published:** 2009-07-21

**Authors:** Hailiang Zhang, Baijun Dong, Jiade J Lu, Xudong Yao, Shilin Zhang, Bo Dai, Yijun Shen, Yao Zhu, Dingwei Ye, Yiran Huang

**Affiliations:** 1 Department of Urology, Fudan University Shanghai Cancer Center, Shanghai, PR China; 2 Department of Urology, Renji Hospital, Shanghai Jiao Tong University School of Medicine, Shanghai, PR China; 3 Department of Radiation Oncology, National University Cancer Institute, National University Health System, National University of Singapore, Singapore; 4 Department of Radiation Oncology, Fudan University Shanghai Cancer Center, Shanghai, PR China

## Abstract

**Background:**

The effects of sorafenib in the treatment of advanced renal cell carcinoma (RCC) have been confirmed in an international collaborative phase III trial. This study aims to confirm similar efficacy and treatment-induced toxicities of sorafenib in the treatment of metastatic RCC in ethnic Chinese patients.

**Methods:**

Ninety-eight consecutive and non-selected patients with pathologically confirmed metastatic RCC were treated according to an institutional treatment protocol. All patients were treated with 400 mg of sorafenib orally twice daily on a continuous basis until disease progression or intolerance to treatment occurred. Dose reduction to 400 mg once daily was required if grade 3 or 4 toxicities occurred. All patients except for 7 received nephrectomy in the course of their disease. All patients were assessed for tumor response, progression-free survival (PFS), overall survival (OS), and treatment-induced toxicities.

**Results:**

The median follow-up time was 76 weeks (range 2–296 weeks) for the entire group of patients. Radiologically confirmed complete response (CR), partial response (PR), stable disease (SD) of more than 4 months, and disease progression as best objective responses were observed in 1 (1%), 23 (23.5%), 62 (63.3%), and 12 (12.2%) patients, respectively. The tumor control rate (CR+PR+SD of >4 months) was 87.8%. The 1-year estimated PFS and OS were 58.4% and 64.6%, respectively. The median progression-free survival (PFS) time was 60 weeks (95% CI 41–79); and the median overall survival (OS) time was not reached with a follow-up of 76 weeks. Reduction of sorafenib dose was required in 26 patients who developed grade 3 or 4 treatment-cause adverse-effects. An additional 9 patients discontinued sorafenib treatment due to severe adverse-effects. No grade 5 toxicity occurred.

Multivariate analysis revealed that independent predictive factors for tumor response to sorafenib treatment included ECOG status, presence of lymph node metastasis, and nephrectomy prior to the development of metastasis.

**Conclusion:**

Sorafenib produced an 87.8% disease control rate for metastatic renal cell carcinoma in Chinese patients, with acceptable rates of toxicity. The medication dosed at 400 mg twice daily is both efficacious and safe in the treatment of metastatic renal cell carcinoma in Chinese patients.

## Background

Renal cell carcinoma (RCC) is the most commonly diagnosed malignancy of the kidney. Although surgery is curative for localized diseases, approximately 30% of patients present with distant metastasis at the time of diagnosis [1]. In addition, more than 25% of patients with locally advanced RCC develop distant metastasis after curative resection. As RCC is highly resistant to chemotherapy, and its response to cytokine therapy including high-dose interleukin-2 (IL-2) and/or interferon-alfa is less than 20% [2,3], the outcome for patients with metastatic disease is dismal: The 5-year overall survival rate despite systemic treatment is less than 10% [4]. Effective systemic treatment for metastatic RCC is clearly needed.

Sorafenib (BAY 43-9006) is a novel agent originally developed as a Raf Kinase inhibitor with a potent effect on C-Raf. Its multi-targeting effects were lately discovered, and in addition to C-Raf, sorafenib also demonstrated effects against B-Raf, vascular endothelial growth factor receptor-2 (VEGFR2), platelet-derived growth factor receptor (PDGFR), Fms-like tyrosine kinase-3 (Flt-3), and stem-cell growth factor (c-KIT) [5]. The efficacy of sorafenib on RCC has been confirmed in both phase II and phase III trials, which had resulted in the approval of its use as a second-line treatment in metastatic disease [6,7]. The progression-free survival (PFS) of patients with advanced RCC reached 5.5 months after sorafenib treatment, as compared to 2.8 months for those received placebo. Sorafenib was approved in most Asian countries/regions including China for metastatic RCC based on these results.

The difference in the expression of tumor markers and molecular features of patients of different ethnic group in a number of malignancies such as lung cancer, prostate cancer, breast cancer, and astrocytoma have been well documented [8-12]. It has also been demonstrated that the RCC diagnosed in different ethnic groups may host different characteristics and behaviors [13]. Although the nature of these variations and their potentially associated molecular basis have not been addressed, it is reasonable to postulate that and the efficacy of sorafenib on advanced RCC may vary in patients of different ethnic background. However, most of the clinical trials of sorafenib for metastatic RCC reported so far included few patients of Asian origin, and the efficacy of sorafenib on RCC diagnosed in Asian patients particularly Chinese has never been reported. The aim of this study is to document the multicenter experience in a relatively large group of Chinese patients with metastatic RCC treated with sorafenib using a protocolized regimen. Special emphasis was placed on the patients' overall and progression-free survivals and treatment-induced toxicities.

## Methods

### Patients and Staging Evaluation

Between March 2006 and March 2008, 98 consecutive and non-selected patients who were diagnosed with metastatic RCC were treated with sorafenib according to an institutional treatment protocol jointly developed by the Departments of Urology of the Fudan University Shanghai Cancer Center and Renji Hospital of Shanghai Jiao Tong University School of Medicine, Shanghai, China, based on the treatment recommendation approved by the U.S. Food and Drug Administration (FDA) for metastatic RCC. The treatment protocol was approved by the institutional review boards of both participating hospitals. All 98 patients had pathologically confirmed RCC from their primary or metastatic site(s), and their tumors were staged according to the AJCC (2002) cancer staging classification as stage IV [14]. Patients with central nervous system metastasis were not eligible to the treatment protocol. Other criteria of exclusion into the treatment protocol included age < 18 or > 80 years, ECOG performance status >3, and life expectancy of less than 3 months.

Pretreatment evaluation consisted of a complete history and physical examination, complete blood count, liver and renal function tests, CT scan of the chest, CT scan or MRI of the abdomen and pelvis, and total body bone scan. FDG-PET or PET/CT scan were optional.

Patient's characteristics are listed in Table 1.

**Table 1:** Patients' characteristics.

Characteristic	No. patients (%)
Age, y	
>= 60	26 (26.5)
<60	72 (73.5)
Sex	
Male	73 (74.5)
Female	25 (25.5)
T-classification at initial diagnosis	
T1	19 (19.4)
T2	30 (30.6)
T3	42 (42.9)
T4	7 (7.1)
AJCC staging group at initial diagnosis	
I	11 (11.2)
II	24 (24.5)
III	18 (18.4)
IV	45 (45.9)
Fuhrman grade	
1	4 (4.1)
2	49 (50.0)
3	39 (39.8)
4	6 (6.1)
Histology	
Predominant clear cell	87 (88.8)
Non-clear cell	11 (11.2)
Sites of metastatic diseases	
Lung	70 (71.4)
Liver	10 (10.2)
Bone	18 (18.4)
Lymph node	24 (24.5)
Adrenal gland	12 (12.2)
Other	12 (12.2)
Number of metastatic foci	
1	20 (20.4)
2	29 (29.6)
3	21 (21.4)
>= 4	28 (28.6)
ECOG performance status	
0	56 (57.1)
1	37 (37.8)
2	4 (4.1)
3	1 (1.0)

### Treatment

Informed consent was required and obtained from all patients prior to the initiation of treatment. All patients received 400 mg of sorafenib orally twice daily, spaced 12 hours apart, on continuous basis. Dose modification to 400 mg once daily was permitted if grade 3 or 4 hematologic toxicity, skin toxicity, hypertension, and/or hepatic dysfunction defined by the NCI-CTCAE 3.0 occurred. The treatment continued until disease progress or intolerance to the treatment occurred. Among all 98 patients, 43 patients initiated their treatment with sorafenib only and 16 received sorafenib with interferon as their first-line therapy, and 39 patients had received sorafenib after they had failed interleukin- and/or interferon-based therapy.

A total of 91 patients had nephrectomy during the course of their disease. Sixty-six patients underwent nephrectomy as part of their definitive treatment for the primary disease prior to the development of metastasis, and 25 patients initially diagnosed with metastatic RCC received nephrectomy for palliative treatment. Seven patients who had metastatic RCC as their initial diagnosis did not receive nephrectomy mostly due to patients' preference.

### Follow-up

All patients were required to be evaluated weekly during their treatment with sorafenib by their attending urologists. Patients also had to be followed-up by their attending urologists every 2 months after the termination of their treatment, if intolerance to the treatment occurred. As a requirement of the protocol, each follow-up session included a complete history and physical examination, routine laboratory tests including CBC, serum electrolytes, liver and renal function tests. CT scan of the chest, abdomen, and pelvis were performed on monthly basis during the treatment, and at every follow-up visit after the termination of the treatment. Tumor response was measured starting at one month after the initiation of the treatment using the RECIST criteria. The radiological responses were evaluated by both diagnostic radiologist(s) independent to this study and verified by investigators.

The adverse-effects secondary to the treatment were evaluated at each visit during and after the treatment, and were recorded according to the Common terminology criteria for adverse events v3.0 (CTCAE) of the National Cancer Institute.

### Data Analysis

Disease control rate was defined as the proportion of patient who achieved stable disease (SD) for more than 4 months, partial response (PR), and complete response (CR) in the entire cohort of 98 cases. Progression-free survival (PFS) time was measured from the date of the initiation of sorafenib treatment until documented radiologically confirmed disease progression or death of patient, whichever is earlier. The duration of overall survival (OS) was calculated from the date of the initiation of sorafenib treatment until death or until the date of the last follow-up visit for patients still alive. Both PFS and OS duration were calculated by the Kaplan-Meier method [15].

## Results

### Treatment outcome

**Figure 1:**
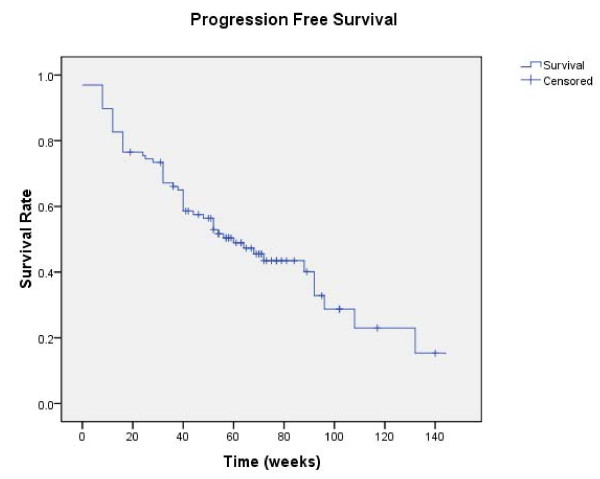
**Progression-free survival of patients**.

**Figure 2:**
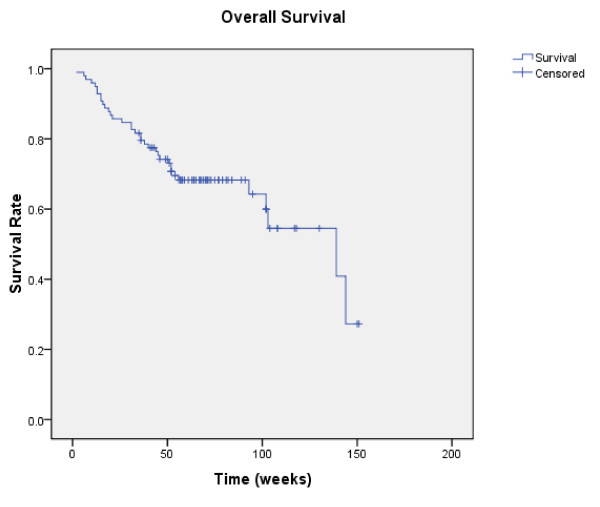
**Overall survival of patients**.

The median follow-up time was 76 weeks (range 2 to 296 weeks) for the entire group of 98 patients. Radiologically confirmed CR, PR, stable disease (of more than 4 months), and disease progression as best objective responses were observed in 1 (1%), 23 (23.5%), 62 (63.3%), and 12 (12.2%) patients, respectively, and the overall disease control rate were 87.8%. The 1-year estimated PFS and OS were 58.4% and 64.6%, respectively (Figure 1 and 2). The median progression-free survival (PFS) was 60 weeks (95% CI 41–79), and the median overall survival (OS) was not reached at the time of this analysis. No statistical differences were observed in OS or PFS for patients received sorafenib as their first-line treatment or after cytokine therapy.

At the time of this analysis, 29 patients (29.5%) had deceased: 24 patients died of disease progression, 4 patients died of intercurrent diseases (heart or pulmonary conditions) that were not associated with their RCC or treatment, and 1 patient died of traffic accident.

### Adverse-Effects

All patients experienced adverse-effects of some sorts; however, the studied treatment protocol was well tolerated and most patients experienced grade 1 or 2 toxicities (Table 2). A total of 26 (26.5%) patients required dose reduction due to grade 3 or 4 adverse-effects, and 9 (9.2%) discontinued their treatment due to severe adverse-effects. The remaining 63(64.3%) patients tolerated and continued their treatment with standard dose of sorafenib at the time of this analysis or until disease progression. The most commonly observed severe toxicity was hand-foot syndrome and anemia, and both were seen in 13 (13.3%) patients. Treatment-induced grade V toxicity was not observed.

**Table 2:** Cumulative incidence of toxicities

	Toxicity grade			
	
	Sorafenib as First Line Treatment (N = 59)		Sorafenib as Second Line Treatment (N = 39)	
	
Toxicity	1–2	3–4	1–2	3–4
Hand-foot syndrome	44	8	21	5
Alopecia	41	1	25	0
Rash	22	0	11	0
Mucositis (oral cavity)	11	1	5	0
Diarrhea	25	1	16	0
Hypertension	14	1	7	1
Fatigue & Anepithymia	41	1	30	2
Weight loss	6	0	3	0
Hemorrhage (nasal				
mucocutaneous)	6	0	3	0
Anemia	7	1	5	0
Thrombocytopenia	0	0	1	0
Leukopenia	0	0	0	0
Hemorrhage (digestive tract)	0	1	1	2
Hematuria	4	0	0	0
Hemoptysis	8	1	4	1
Liver dysfunction	7	2	3	0
Angina	0	1	0	0

Table 2 detail the cumulative incidence of toxicities observed in the entire group of patients.

### Predictive Factors of Response to Sorafenib

The value of various potential factors including patients' age, gender, ECOG status before treatment, pathologic type, tumor differentiation, presence of lymphvascualr invasion, number of metastatic foci (1–3 versus >3), and timing of sorafenib treatment (first-line versus second-line) were evaluated in both uni- and multivariate analyses for predicting the response to sorafenib. Univariate analyses indicated that pretreatment ECOG status, presence of lymph node metastasis (N+), number of metastatic foci (1–3 versus >3), and nephrectomy as primary treatment prior to the development of distant metastasis were significant predictive factors of tumor control (CR, PR, or stable disease of more than 4 months) to sorafenib treatment. However, only favorable pretreatment ECOG status, N+, and the use of nephrectomy were statistically significant for predicting the disease control on multivariate analyses (Table 3).

**Table 3:** Multivariate analysis

Predictive Factors	CR+PR+SD	PD	Number (%)	X^2^	P
Lymph node metastasis at initial diagnosis				4.69	0.030
N0	44(60.27)	29(39.73)	73(100.00)		
N+	21(84.00)	4(16.00)	25(100.00)		

Nephrectomy before metastasis				8.05	0.005
No	15(46.88)	17(53.12)	32(100.00)		
Yes	50(75.76)	16(24.24)	66(100.00)		

ECOG				11.28	0.010
0	44(78.57)	12(21.43)	56(100.00)		
1	20(54.05)	17(45.95)	37(100.00)		
2	1 (25.00)	3(75.00)	4(100.00)		
3	0(0.00)	1(100.00)	1(100.00)		

## Discussion

Renal cell carcinoma (RCC) is the predominant form of kidney cancer, and systemic therapy using cytokine or cytotoxic agents provides limited effects to RCC with distant metastasis. An international collaborative study has confirmed that targeted therapy agent sorafenib (BAY 43-9006) was effective in the treatment of metastatic RCC and could provide a significant improvement in the progression-free survival (PFS) with acceptable adverse-effect profile [7]. In the current study, we have demonstrated that sorafenib is equally effective in the treatment of metastatic RCC diagnosed in patients of Chinese ethnic background. The median progression-free survival time was 60 weeks, and overall survival time was not reached after a follow-up of 76 weeks. In addition, sorafenib was well tolerated by Chinese patients with metastatic RCC: Grade 3 and 4 toxicities were observed in 35.7% patients, and only 9 (9.2%) patients terminated their treatment due to adverse-effects. Furthermore, disease control, which was defined as radiologically confirmed CR, PR, and stable disease of more than 4 months, was achieved in 87.8% of patients. Results of multivariate analysis revealed that presence of lymph node metastasis, better ECOG status, and use of nephrectomy for definitive treatment prior to the development of metastatic disease were significant factors for predicting disease control to sorafenib treatment.

Sorafenib is a multikinase inhibitor and has potent effects on c-Raf, b-Raf, VEGFR-2, and PDGFR, and it is postulated that the effect of sorafenib on RCC is closely associated with its effect on VEGF receptor [16]. The exact mechanism of its effect on RCC is largely unknown, but its efficacy on metastatic RCC as a second-line treatment has been proven. Although variations in the expression of VEGF receptors and other potential treatment targets of RCC in patients of different ethnic background have not been reported, race-dependent variation in other molecular targets such as epidermal growth factor (EGF) is well known. For example, gefitinib is a tyrosine kinase inhibitor that has been actively utilized in the treatment of non-small-cell lung cancer (NSCLC) particularly in Asia. Its efficacy in NSCLC has been repeatedly demonstrated in Asian patients; however, the results of an international collaborative study did not confirm such findings [17], and the medication was not approved for the treatment of NSCLC in the United States by the Food and Drug Administration. It was lately discovered that the efficacy of gefitinib was associated with EGF mutations, which is significantly more prevalent in Asian patients with NSCLC, particularly adenocarcinoma [18,19], thus explained the discrepancy in the outcome of NSCLC after gefitinib treatment. Similar findings have been reported in the expression of other molecular targets for treatment in various types of malignancies such as breast cancer diagnosed in patients with different ethnic background [8-12]. In the case of renal cell carcinoma, it has been demonstrated that the malignancy diagnosed in various ethnic groups had different clinical characteristics: The presenting symptoms, the course of disease, and the outcome after standard treatment varied significantly between patients of Caucasian, Hispanic, African-American, and Asian backgrounds [13]. Whether these differences were induced by any variations at molecular level remained unknown; however, it is reasonable to postulate that the differences in the tumor cell at the genetic level play an important role in determine the differences in the phenotype of the disease, and question whether sorafenib provides similar level of efficacy in Asian patients with metastatic RCC. The results of the current study revealed that sorafenib produced a median progression-free survival time of 60 weeks and was well tolerated. As treatment options for metastatic RCC is relatively limited, and response of RCC to chemotherapy or immunotherapy is suboptimal, our favorable results which confirmed the efficacy of sorafenib in Asian particular Chinese patients supported practice change in the treatment of metastatic RCC.

Reports on the efficacy of sorafenib in non-Caucasian patients with advanced RCC have been limited. In a non-planned subgroup analysis of 15 Spanish patients accrued in the TARGET study (7 on sorafenib arm and 8 on placebo arm), a trend of improved PFS was demonstrated, and the author concluded that the efficacy and toxicity of Spanish patients follow the trend observed for the overall international population [20]. In addition, a phase II clinical trial from Japan confirmed the efficacy of sorafenib in Japanese patients with advanced RCC who had nephrectomy and had failed cytokine-containing therapy. The study focused on the response to treatment as well as PFS, and reported partial response and stable disease (of more than 4 weeks) rates of 14.7% and 72.1%, respectively, with a total disease control rate of 86.8%. The PFS of the entire group of patients was 224 days (32 weeks) [21]. The overall disease control rate in the current series was 87.8%, which was similar to the finding of the Japanese study. In addition, the median PFS of 60 weeks from our series also provided a reasonable support to the Japanese data and the use of sorafenib in Chinese patients with advanced RCC.

Despite of the prospective nature of our treatment protocol and its favorable results, several important issues need to be addressed in the current study. First of all, the current study is a single arm series aimed to study the efficacy of sorafenib in the treatment of metastatic RCC in Chinese patients. Although all patients were treated with sorafenib according to a prospectively designed regimen, the study was not designed as a prospective phase II trial. Sorafenib was approved by the State Food and Drug Administration (SFDA) of China for its use in patients with advanced RCC, further investigation for a proven medication in the form of a prospective phase II or randomized clinical trials would not be easily acceptable by patients with a terminal disease, considering sorafenib was the only approved effective treatment for advanced RCC in China when we initiated this study.

In addition, a substantial portion of the patients our series were cytokine naïve and received sorafenib as the first-line treatment. While phase II and III trials have repeated demonstrated the effect of sorafenib on advanced renal cell carcinoma, treatment in the majority of previously reported trials were delivered after the failure of cytokine treatment. Although no significant difference was found among patients received first-versus second-line treatment in our trial, the small number of both subgroups precluded a statistically meaningful comparison. In addition, the mixed timing of sorafenib use might adversely affect the uniformity of the treatment outcome. The effect of sorafenib as a first-line treatment in advanced RCC comparing to conventional therapy, i.e., nephrectomy followed by cytokine therapy has not been confirmed. And whether a progression-free survival or overall survival benefit can be further provided by early use of sorafenib therapy particularly in Chinese patients remains unknown. Thirdly, concurrent therapy using cytokine with sorafenib was not prohibited in the protocol. Among all patients received sorafenib as the first-line treatment, 16 patients received concurrent cytokine treatment. Results from a phase I study indicated that sorafenib plus interferon was well tolerated [22]; furthermore, sorafenib plus interferon, either as first- or second-line treatment for advanced RCC produced substantial activity in patients with advanced RCC, as reported in two phase II trials [23,24]. However, comparison of patients treated with or without interferon in our series is not feasible as the number of patients was limited. We consider the non-protocolized treatment with cytokine in this small portion of patients as well as the design of the study (i.e., non-phase-II trial) served as two pitfalls of our series.

Multivariate analyses of the current study showed that ECOG, presence of lymph node metastasis (N+), and use of nephrectomy during the course of treatment were significant predictive factors for disease control by sorafenib. Although favorable ECOG status may assure better tolerance to the treatment, the association of nodal metastasis and use of nephrectomy to improved response to sorafenib requires further discussion. Results from preclinical studies have indicated that VEGFR is associated with hematogenous metastasis in renal cell carcinoma [25,26], and tumor metastasis via lymphatics can be inhibited by interference with the VEGF-C VEGFR3 interaction [27]. It is possible that the postulated targets of sorafenib such as VEGFR are also associated to nodal metastasis in RCC. However, further investigation is needed to confirm such association. Furthermore, nephrectomy followed by Interferon Alfa-2b was associated with significant improvement in medial survival as compared to those treated with interferon alone, and the median survival were 11.1 months versus 8.1 months for patients treated with or without nephrectomy [28]. However, the molecular basis of the association between nephrectomy and improved patients' prognoses is unknown. In the current study, patients who had completed nephrectomy for definitive treatment prior to the development of metastasis experienced improved response to sorafenib, as compared to those who received nephrectomy for their metastatic disease and those who did not receive surgery combined. In other words, it appears that metastatic foci developed after the resection of the primary disease responded to sorafenib better than those developed with the primary disease intact. Although the clinical and molecular bases of the findings in the multivariate analyses are unknown, these results are important clinically and certainly warrant further study for its root cause. Further investigation for establishing a predictive model for individualizing sorafenib use in advanced RCC should also be considered.

The median PFS of our series was 60 weeks, and the median OS was not reached. The underlying reason of this seemingly more favorable outcome as compared to the results of TARGET study and the phase II trial from Japan is unknown at this time. The outcome between the three studies cannot be compared directly; however, the magnitude of the discrepancy in PFS warrants further exploration and confirmation. One potential explanation of such discrepancy is the inherent differences between ethnic Chinese and other ethnic groups. In a pooled safety analysis of sorafenib in the treatment of solid tumors including RCC, severity of skin reaction and diarrhea was significantly associated to time-to-progression [29]. It is interesting to note that the probabilities of hand-foot skin reaction (HFSR) in our series and in the sorafenib Asian-Pacific trail on hepatocellular carcinoma were substantially higher [30], as compared to the TARGET study and the European SHARP trail [7,31]. It seems that Asian particularly Chinese patients are more prone to HFSR caused by sorafenib. However, whether the ethnic background and its associated molecular mechanism serves the major difference in the efficacy of sorafenib in RCC subject to further investigation. Furthermore, since sorafenib was used as a first-line treatment in the majority of our patients, with or without concurrent cytokine treatment. Whether timing for the delivery of medication had affected the treatment outcome in Chinese patients should be considered. In addition, as the exact mechanism of sorafenib on RCC remains unknown, investigation of any phenotypic variations is currently not feasible. One of the possible solutions is to utilize gene-profiling technology to detect the differences between patients with different response to the treatment. In the participating hospitals of the current study, tumor tissues from patients accrued have been harvested and stored for a parallel study under planning.

## Conclusion

Sorafenib was efficacious in the treatment of metastatic renal cell carcinoma in Chinese patients, and was well tolerated. The median progression-free survival time was 60 weeks, and overall survival time was not reached after a follow-up of 76 weeks. Multivariate analyses revealed that favorable ECOG status, presence of lymph node metastasis, and nephrectomy prior to the development of metastasis were significant predictive factors of tumor response to sorafenib.

## Competing interests

The authors declare that they have no competing interests.

## Authors' contributions

HZ, BD and JL carried out the review of literature and drafted the manuscript. HZ and JL performed the statistical analysis. DY and YH designed the treatment protocol and the study, and helped to with statistical analysis. XY, SZ, BD, YS, and YZ participated in the study design, performed the data collection and chart review, and helped with the draft of the manuscript.

## Pre-publication history

The pre-publication history for this paper can be accessed here:

http://www.biomedcentral.com/1471-2407/9/249/prepub
